# Cell-Based Sensors for the Detection of EGF and EGF-Stimulated Ca^2+^ Signaling

**DOI:** 10.3390/bios13030383

**Published:** 2023-03-14

**Authors:** Euiyeon Lee, Keshab Lal Shrestha, Seonhye Kang, Neethu Ramakrishnan, Youngeun Kwon

**Affiliations:** 1 Department of Biomedical Engineering, Dongguk University, Seoul 04620, Republic of Korea; 2 Department of Chemistry and Chemical Biology, Rutgers University, Piscataway, NJ 08854, USA

**Keywords:** cell-based biosensor, split-inteins, conditional protein cleavage, signal peptide, epidermal growth factor receptor

## Abstract

Epidermal growth factor (EGF)-mediated activation of EGF receptors (EGFRs) has become an important target in drug development due to the implication of EGFR-mediated cellular signaling in cancer development. While various in vitro approaches are developed for monitoring EGF-EGFR interactions, they have several limitations. Herein, we describe a live cell-based sensor system that can be used to monitor the interaction of EGF and EGFR as well as the subsequent signaling events. The design of the EGF-detecting sensor cells is based on the split-intein-mediated conditional protein trans-cleavage reaction (CPC). CPC is triggered by the presence of the target (EGF) to activate a signal peptide that translocates the fluorescent cargo to the target cellular location (mitochondria). The developed sensor cell demonstrated excellent sensitivity with a fast response time. It was also successfully used to detect an agonist and antagonist of EGFR (transforming growth factor-α and Cetuximab, respectively), demonstrating excellent specificity and capability of screening the analytes based on their function. The usage of sensor cells was then expanded from merely detecting the presence of target to monitoring the target-mediated signaling cascade, by exploiting previously developed Ca^2+^-detecting sensor cells. These sensor cells provide a useful platform for monitoring EGF-EGFR interaction, for screening EGFR effectors, and for studying downstream cellular signaling cascades.

## 1. Introduction

Interactions between the epidermal growth factor (EGF) family and the EGF receptor (EGFR) tyrosine kinase family play a key role in modulating cell proliferation and differentiation [1,2,3]. Once EGF binds to an EGFR, it induces conformational changes that lead to the activation of the tyrosine kinase domain and the downstream signaling cascade to control cell proliferation, apoptosis, and angiogenesis. In particular, the significance of EGFR signaling cascade in cancer progression has been drawing substantial attention; the EGF stimulation system for growth regulation is implicated in both normal and neoplastic cell proliferation [2,4,5,6]. The roles of EGF and EGFR in human cancer have been extensively reviewed with an emphasis on their clinical significance including consideration of multiple inhibitory strategies targeting EGFR activity for cancer therapeutics [3,7,8]. Especially, controlling the signaling pathways involving the EGFR family is expected to provide critical opportunities for developing molecular target strategies for cancer therapy [5,9,10,11,12].

In this aspect, bioanalytical tools for investigating the interaction between EGFR and its targeting ligand offer critical opportunities in drug screening. Conventionally, various in vitro approaches such as affinity chromatography, enzyme-linked immunosorbent assay (ELISA), and surface plasmon resonance (SPR) are used for detecting EGF and EGFR interactions [13,14]. While these approaches can be used for quantitative analysis, they suffer from limitations as they require the use of purified membrane proteins. Purification of purified membrane receptors is not only expensive but also frequently causes a loss of activity compared to that in their native environment, thereby causing inaccuracy in the assay results to limit their usage. Alternatively, cell-based sensing approaches can be exploited to investigate EGFR homodimerization using optical detection methods such as single molecule tracking and fluorescence correlation spectroscopy [15,16,17,18]. Although they offer a highly sensitive investigation of the interactions and dynamics of biomolecules, they are not suitable for monitoring the various effectors of EGFR as this type of study often requires measuring the ensemble average of the binding event.

Cell-based optical biosensors have emerged as a powerful tool for screening biological effectors for various receptors as they can monitor biological interactions in their native context where their biological actions are taking place [19,20,21,22,23,24,25,26]. Sensor cells are generated by genetically encoding sensor proteins that contain a molecular recognition element as well as a reporter element. A native receptor or enzyme is frequently used as the recognition element and an optically active protein such as an auto-fluorescent protein (AFP) or a luciferase is used as the reporter element. The reporter element conveys the presence of the target via fluorescence activation/deactivation, fluorescent/bioluminescent resonance energy transfer (FRET/BRET), and bimolecular fluorescence/luminescence complementation (BiFC/BiLC) [27,28,29,30,31,32]. Although FRET-based detection is a plausible tool for monitoring EGFR dimerization, this approach often suffers from false positive signals coming from a high concentration of AFPs located nearby as well as weak signal intensity due to interference by external factors that cause low FRET efficiency [27,29,33,34,35,36,37,38].

To overcome these limitations, we designed a cell-based sensor for the detection of EGF-EGFR interaction using a reporting strategy based on intein-mediated reconstitution of the signal peptide, and consequent fluorescence translocation. Intein (a self-processing protein) is utilized to form or break specific amide bonds to activate the signal peptide [39,40,41]. Split-intein-mediated conditional protein splicing (CPS) or conditional protein cleavage (CPC) reaction is initiated by the presence of target molecules as a trigger [42]. The activated signal peptide then translocates the fluorescence cargo to the target cellular compartment in order to report the presence of the target molecule.

**Scheme 1:**
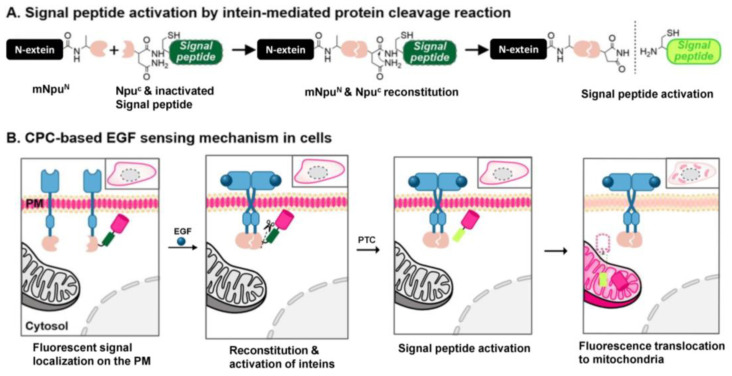
A schematic representation of epidermal growth factor (EGF)-detection mechanism in sensor cells. (**A**) The general concept of signal peptide activation via the intein-mediated cleavage reaction. (**B**) Design and working mechanisms of conditional protein cleavage (CPC)-based EGF sensing in live cells. EGF triggers split-intein reconstitution and activation. The split intein-mediated CPC reaction is used to cleave and thereby activate the signal peptide. Finally, fluorescent reporter mCherry is translocated to the mitochondria for reporting the presence of target molecule EGF.

In the present study, we exploited this signal peptide activation and fluorescence translocation strategy in constructing a sensor cell for detecting the presence of EGF, by employing a mitochondrial-targeting sequence (MTS) as an activatable signal peptide and EGFR as a target receptor (Scheme 1A). An EGF-detecting sensor cell was fabricated by exploiting the intein-mediated CPC reaction that can free and activate the MTS introduced as a C-extein of the split C-intein (Int^C^) (Scheme 1A,B). A mutation was introduced to one of the penultimate residues of the split N-intein (Int^N^) to induce intein-mediated protein cleavage instead of protein splicing. The presence of the target ligand for EGFR-induced dimerization to switch on the intein-mediated cleavage reaction, and consequentially to activate MTS. The translocation of the fluorescence signal to the mitochondria was monitored in the presence of target ligand EGF in the sensor cells. The sensitivity and response time of sensor cells were investigated for the performance analysis and then sensor cells are used for screening agonist and antagonist of EGFR. We also demonstrated the use of the cell-based sensor technology for monitoring subsequent Ca^2+^ signaling induced by the EGF-EGFR interactions.

## 2. Materials and Methods

### 2.1. Materials and Microscopic Imaging Apparatus

All of the chemicals at the best grade available were purchased from Sigma-Aldrich (St. Louis, MO, USA) and Fisher Scientific (Pittsburgh, PA, USA) unless otherwise stated. DNA oligonucleotides were acquired from MBiotech (Hanam, Republic of Korea) and restriction enzymes were purchased from Elpis Biotech (Daejeon, Republic of Korea) and New England Bio Labs (Ipswich, MA, USA). Cell culture reagents were procured from Invitrogen Life Technologies (Carlsbad, CA, USA) and Welgene (Daegu, Republic of Korea). Cetuximab was purchased from MedChemExpress (Monmouth Junction, NJ, USA) and transforming growth factor-α (TGF-α) was purchased from Acro Biosystems (Cambridge, MA, USA).

Confocal fluorescence microscopy images were obtained using an Eclipse Ti (Nikon Instruments, Tokyo, Japan) with excitation wavelengths of 358, 488, and 594 nm using the corresponding filters. Fluorescence in the images was also visualized using a fluorescence microscope (Zeiss Axio Imager Z2, Jena, Germany); the fluorescence intensity was measured using Nikon NIS-Element BR 4.60 software and quantitative analysis was performed using Image J ver.1.53 (NIH, Bethesda, MD, USA).

### 2.2. Plasmid Construction

DNA cloning was carried out according to standard protocols. All of the constructed plasmids were DNA-sequenced, amplified using Escherichia coli strain DH5α, and then used for protein expression. The cDNA encoding the N-terminal domain of Nostoc punctiforme (*Npu*) DnaE split-inteins (Npu^N^) was inserted between NotI and HindIII in pBI-CMV1. To construct mutated and nonfunctioning Npu^N^ (mNpu^N^), site-directed mutagenesis was carried out to change the first amino acid of Npu^N^, cysteine, to alanine using a QuikChange Site-Directed Mutagenesis Kit (Agilent, Santa Clara, CA, USA) according to the vendor’s protocol. EGFR was introduced to the C-terminus of mNpu^N^ between MluI and NotI to create construct **1**, mNpu^N^-EGFR. The cDNA encoding EGFR (Addgene plasmid 32751) was obtained from Addgene (Cambridge, MA, USA). The cDNA encoding the C-terminal domain of *Npu* DnaE split-inteins (Npu^C^) in pET28a was modified by introducing cDNA rMTS, *CFNGS*MLSLRQSIRFFKPATRTLCSSRYLL, to the C-terminus between the BamHI and SacI sites. rMTS sequence include MTS (MLSLRQSIRFFKPATRTLCSSRYLL) derived from the cytochrome c oxidase subunit 4 (COX4) from Saccharomyces cerevisiae and additional *CFNGS* sequence to facilitate intein-mediated reaction. Then, cDNA encoding mCherry was inserted in the C-terminus between SacI and SalI. The resulting construct was modified by inserting EGFR on the N-terminus between NcoI and NheI to create a construct encoding fusion protein **2**, EGFR-Npu^C^-rMTS-mCherry. This construct was introduced into pBI-CMV1 containing construct **1** using restriction enzyme sites AgeI and XbaI to express fusion proteins **1** and **2** together. The cDNA encoding the rMTS and mCherry were introduced into MCS2 in pBI-CMV1 using restriction enzyme sites AgeI and XbaI to create construct **3**. The N36A point mutation of Npu^C^ was introduced into pBI-CMV1 containing constructs **1** and **2** to generate EGFR-mNpu^C^-MTS-mCherry **4**.

### 2.3. Live Cell Imaging for EGF and Ca^2+^ Sensing

For live cell imaging, HeLa cells were grown in 35 mm confocal imaging dishes. The cells were transiently transfected using the pBI-CMV1 vector encoding fusion proteins **1** and **2**. Gene expression was allowed to proceed at 37 °C for 48 h, after which cell-based sensing assays were started. For EGF sensing, live cells were treated with 100 ng/mL EGF for a given time period. Afterward, cell mitochondria were stained using MitoTracker Green (Thermo Scientific, Waltham, MA, USA) or CytoPainter MitoBlue (Abcam, Cambridge, UK) according to the vendor’s protocol, washed twice using phosphate-buffered saline (PBS). Approximately 10 to 20 cells were analyzed in each experiment and the entire cell area was analyzed for colocalization. Co-localization was quantified by using the Pearson’s correlation through the JaCoP Plug-in on ImageJ. The *R* value was scored from 1 to −1, with 1 standing for a completely positive correlation and −1 for a negative correlation, with zero standing for no correlation. For analysis of Ca^2+^ sensing, fluorescence intensity of each cell compartment was analyzed using ImageJ software. The red fluorescence intensity ratio (Nuc/Cyto) was calculated based on the fluorescence images using ImageJ software. The red fluorescence signal intensity in the nucleus and cytoplasm were determined, first, by drawing a region of interest (ROI) occupying approximately 50% of the area and then measuring the average pixel intensity of the ROI.

### 2.4. Western Blot Analysis

For Western blot analysis, HeLa cells (2 × 10^6^) were grown in 100 mm dishes, transfected with plasmid DNA containing fusion proteins **1** and **2**, and treated with analytes for 1 h. Cells were collected and lysed on ice using 500 µL of RIPA lysis buffer (25 mM Tris-HCl pH 7.6, 150 mM NaCl, 1% NP-40, 1% Sodium deoxycholate, 0.1% SDS) (Thermo Scientific, Waltham, MA, USA) containing a protease inhibitor cocktail. Cell lysates were mixed with 0.2 volumes of 5× sodium dodecyl sulfate (SDS)-polyacrylamide gel electrophoresis (PAGE) loading buffer and then 20 μL containing approximately 16 μg protein was subjected to SDS-PAGE. Then, SDS-PAGE gels (10% bis-tris) were transferred to the PVDF membrane and blocked with 2% *w/v* BSA in TBST (25 mM Tris, 150 mM NaCl, 0.1% *v/v* Tween-20, pH 7.6) at room temperature for 1 h. The formation of the cleavage product was analyzed by staining with anti-mCherry antibodies (Abcam, Cambridge, UK) and goat anti-rabbit IgG/HRP (Abcam, Cambridge, UK). Protein analysis was carried out by using a West Pico PLUS Chemiluminescent Substrate (Thermo Scientific, Waltham, MA, USA) on an ImageOuant LAS 500 imaging system (GE Healthcare, Chicago, IL, USA).

### 2.5. Statistical Analysis

All data are presented as the mean ± standard error of the mean. A comparison between different groups was carried out using a one-way analysis of variance (ANOVA) followed by a Tukey’s multiple-comparison test. *p* < 0.05 was considered to be significant. Co-localization analysis was performed using Pearson’s correlation coefficient (*r*) in the ImageJ plugin JACoP. Graphs were plotted using GraphPad Prism 8.0 (Graphpad, San Diego, CA, USA).

## 3. Results and Discussion

### 3.1. The Design and Construction of Sensor Proteins for Detecting Ligand-Induced EGFR Dimerization

**Figure 1:**
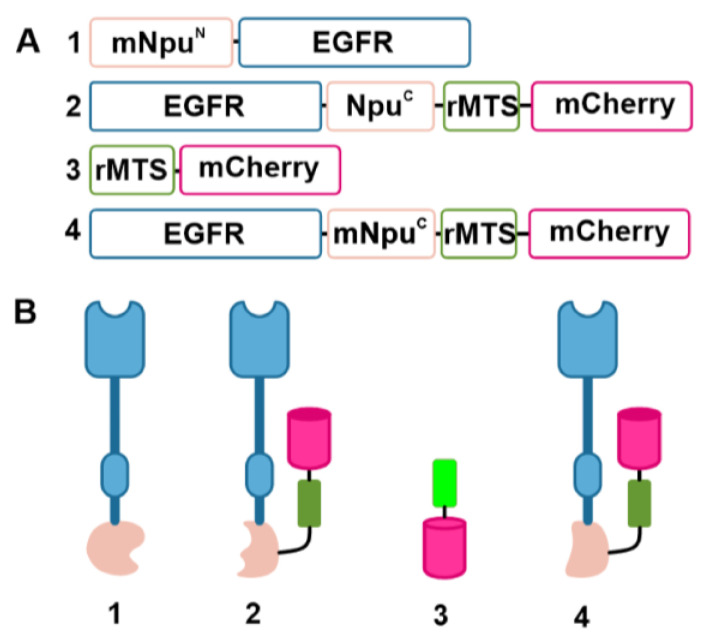
The fusion proteins used in this study. (**A**) Domain architecture of the DNA constructs and (**B**) a schematic illustration of fusion proteins 1, 2, 3, and 4. EGFR, epidermal growth factor receptor; rMTS, recombinant and modified mitochondrial targeting sequence; mNpu^N^, the C1A mutant N-terminal domain of *N. punctiforme* (Npu) intein; Npu^C^, the C-terminal domain of Npu intein; mNpu^C^, the N36A mutant of C-terminal domain of Npu intein.

A CPC system that is triggered by the presence of EGF was designed for constructing the EGF-detecting cell-based sensor. The domain structure and a schematic illustration of the fusion proteins are shown in Figure 1A,B, respectively. Target receptor EGFR was selected as a recognition element as it binds to EGF, then goes through a detectable conformational change. Fast-reacting *Npu* DnaE split-inteins Npu^N^ and Npu^C^ were chosen to mediate the protein trans-cleavage reaction. [43,44,45] Although *Npu* DnaE split-inteins are rarely used for the CPC or CPS reaction as they react spontaneously in solution without external stimuli, we anticipated that anchoring them to the plasma membrane would block their spontaneous reconstitution, which would enable CPC activation upon the homodimerization of EGFR. We also introduced the C1A mutation to Npu^N^ to generate mNpu^N^, which can be induced to mediate protein cleavage by blocking the protein splicing mechanism. The modified MTS sequence (rMTS), *CFNGS*MLSLRQSIRFFKPATRTLCSSRYLL, in which *CFNGS* was introduced to the N-terminus of the MTS sequence originating from *S. cerevisiae* COX4 to ensure the procession of the intein-mediated cleavage reaction. For conditional protein cleavage by intein to be effective, CFN are essential amino acids. An additional amino acid, GS is produced from molecular cloning process to insert Npu^C^. It was selected as a signal peptide that is activated when separated from the membrane protein [46]. Mitocondrial localizing capability of rMTS was estimated using the prediction program Deeploc [47] (Figure S1). Fusion proteins **1** and **2** were prepared to construct a sensor cell. Fusion protein **1,** mNpu^N^-EGFR, was created by introducing mNpu^N^ to the N-terminus of EGFR and fusion protein **2,** EGFR-Npu^C^-rMTS-mCherry, was created by first introducing Npu^C^ to the C-terminus of EGFR, then rMTS to the C-terminus of Npu^C^ as C-extein, and finally, mCherry to the C-terminus of rMTS as the optical cargo. When fusion proteins **1** and **2** coexist in the plasma membrane, the EGF-induced dimerization of EGFR prompts reconstitution of split-*Npu* inteins to initiate the CPC reaction. Subsequently, rMTS is freed from the plasma membrane to travel to the mitochondria with the fluorescent cargo attached to its C-terminus (Scheme 1B). The gene construct encoding the CPC product, rMTS-mCherry **3**, was cloned separately to evaluate the mitochondrial targeting ability of rMTS. HeLa cells expressing fusion protein **3** showed that rMTS can deliver an AFP to the mitochondria (Figure S1). In addition, the N36A mutation was introduced to Npu^C^ of fusion protein **2** to produce nonfunctional mock sensor protein **4** without the intein-mediated cleavage or splicing ability.

### 3.2. Generation and Evaluation of the EGF-Detecting Sensor Cells

**Figure 2:**
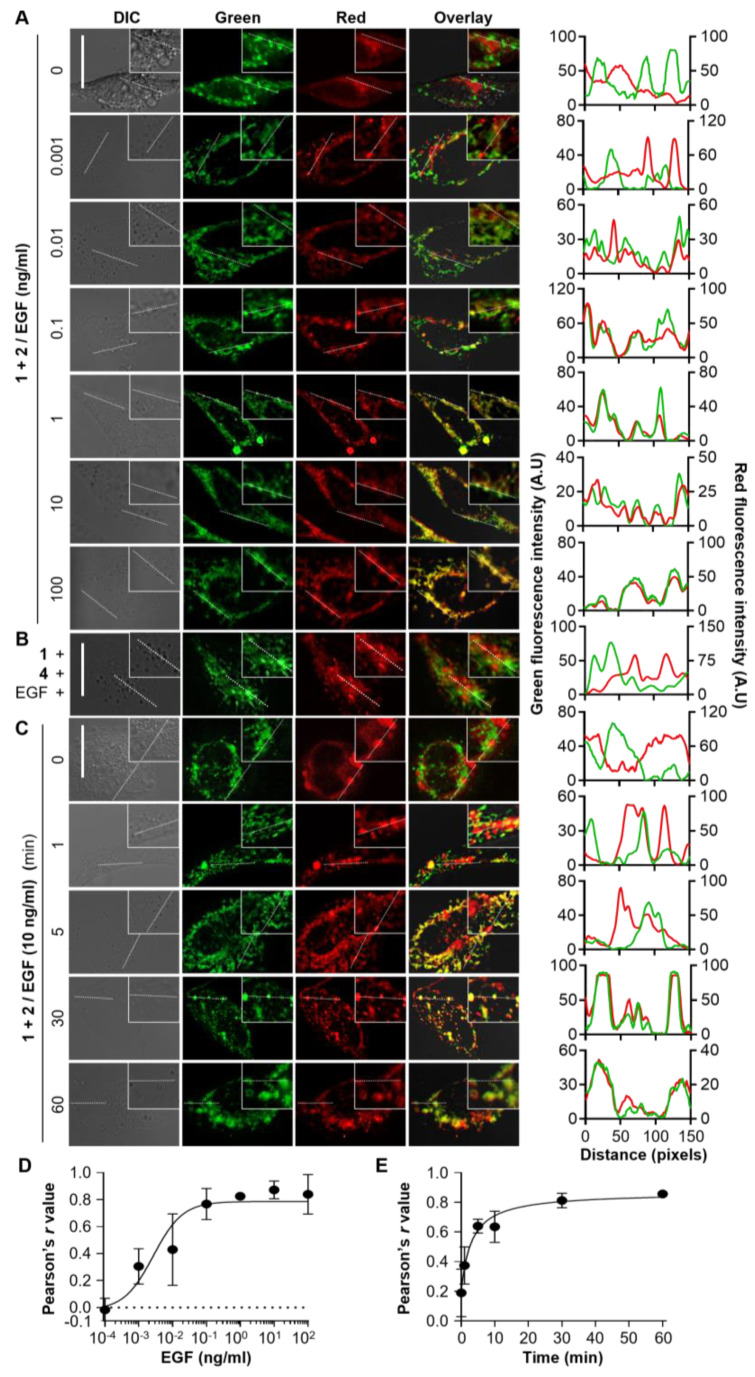
Performance analysis of sensor cells at different EGF-dose and EGF-treatment time. Fluorescence images of (**A**) sensor cells, expressing fusion proteins **1** and **2**, treated with various concentrations of EGF (scale bar = 20 μm) and (**B**) mock sensor cells, containing fusion proteins **1** and **4**, challenged with EGF (10 ng/mL) (scale bar = 20 μm). Fluorescence translocation was observed from functioning sensor cells treated with EGF at concentrations higher than 0.1 ng/mL. (**C**) Fluorescence images of sensor cells treated with EGF for various time points. The minimum response time was 5 min. Plots of the red and green fluorescence signal co-localization versus (**D**) varying EGF concentration or (**E**) varying exposure time. Approximately 10 to 20 cells were analyzed in each experiment and the entire cell area was analyzed for colocalization. Co-localization was quantified by using Pearson’s correlation through the JaCoP Plug-in on ImageJ. The *R* value was scored from 1 to −1, with 1 standing for complete positive correlation and −1 for a negative correlation, with zero standing for no correlation.

The EGF-detecting sensor cells were generated by transfecting HeLa cells using plasmid encoding fusion proteins **1** and **2** with constitutive promoters. The performance of the sensor cells was evaluated by treating them with signaling molecule EGF at various concentrations ranging from 0.001–100 ng/mL for 1 h. The sensor cells were stained with mitochondrial-staining dye, then translocation of mCherry to the mitochondria was analyzed via fluorescence co-localization analysis using ImageJ. The results show that the sensor cells were responsive to target EGF in a dose-dependent manner (Figure 2A,D). The red fluorescence signal was spread out in the cytosol with a weak localization pattern in the membrane fraction in non-treated control sensor cells and cytosolic signal did not correlate with the mitochondrial location (Figure 2A, first lane). We supposed the red fluorescence signal seemed to appear in the cytoplasm of the sensor cells in the absence of EGF because EGFR is located in various subcellular locations including the cytoplasm and the nucleus as previously reported [48,49], and paid attention to mitocondrial localization of signal to detect EGF. The red fluorescence signal started to show weak correlation with the green mitochondrial-staining signal at EGF concentrations from 0.001 to 0.01 ng/mL and showed a strong correlation from 0.1 ng/mL upward. Pearson’s *r* for correlation was 0.08 for non-treated control sensor cells and were 0.06, 0.18, 0.76, 0.83, 0.87, and 0.84 for 0.001–100 ng/mL EGF-treated sensor cells, respectively. The sensor cells exhibited excellent sensitivity with a limit of detection (LOD) of 0.1 ng/mL. This level of sensitivity has been previously reported when the assay was carried out using purified model systems, which further signifies our result [50,51] (Figure 2B), indicating that the observed fluorescence translocation in the sensor cells can be attributed to the split intein-mediated CPC.

A fast response time is critical for the practicability of an analytical sensor. To estimate that of the developed sensor cells, we studied their time response after treating them with EGF (10 ng/mL) for various time periods from 1 to 60 min. Cells were washed with PBS after the given period of exposure time, stained using mitochondrial-staining dye, and then observed using fluorescence microscopy for co-localization analysis. The results show that the sensor cells started to show translocation and consequential co-localization as early as 5 min after treatment with EGF, with maximal co-localization after 30 min (Figure 2B,E). These results together demonstrate the superb performance of the developed sensor cells in terms of high sensitivity and a fast response time.

### 3.3. Detecting an Agonist and Antagonist of EGFR Using the Genetically Encoded EGF Sensor Cells

Following the validation of our cell-based EGF sensing system, we sought to further broaden its utility by using it to detect agonists and antagonists of EGFR. Both EGF and EGFR are often constitutively stimulated in many cancers, thus inhibitors for EGF binding to EGFR are popular drug candidates. As the developed sensor cells enable the screening of analytes based on their biological activity, which is unlike immuno-sensors that screen analytes based on their structure, the sensor cells provide a suitable platform for screening agonists and antagonists of EGFR.

**Figure 3:**
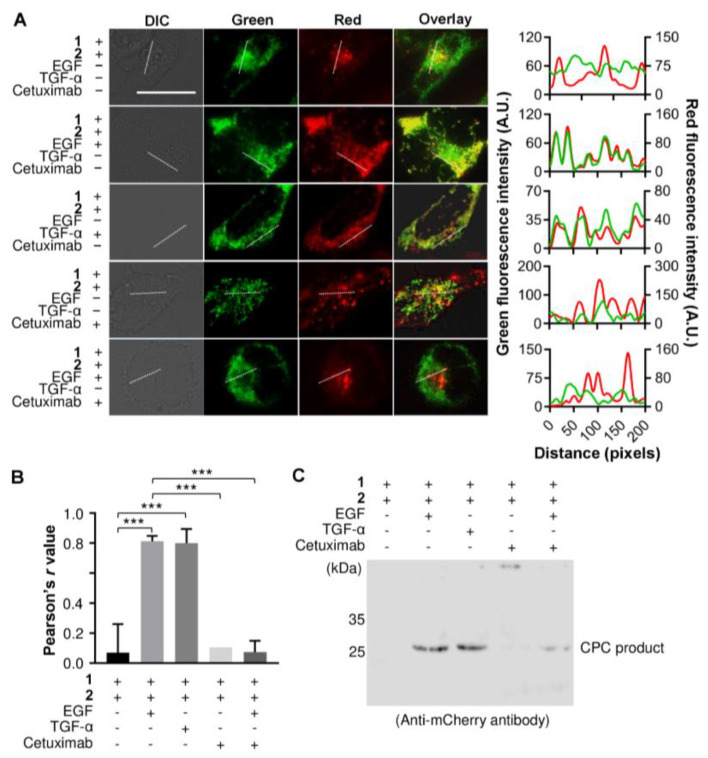
Screening of agonist and antagonist using the sensor cell. (**A**) Fluorescence image of sensor cells when non-treated (row 1), or treated with EGF (row 2), TGF-α (100 ng/mL, row 3), Cetuximab (400 nM, row 4), or Cetuximab/EGF (row 5), individually. Red and green signal colocalization was observed when sensor cells were treated with EGF or an agonist, TGF-α. (scale bar = 20 µm). Cetuximab did not induce signal translocation and repressed EGF-induced translocation. (**B**) The level of signal colocalization is reported in Pearson’s *r* value for sensor cells treated with various effectors. Statistical analysis was carried out via one-way ANOVA followed by a Tukey’s multiple comparison test (*** *p* < 0.001). (**C**) Western blot results showing the formation of the conditional protein cleavage product in the presence of the target EGF or an agonist TGF-α.

TGF-α and Cetuximab were selected as the representative agonist and antagonist, respectively. TGF-α (a member of the EGF family) was chosen as it is one of the most widely studied EGFR agonists [52], while Cetuximab is a monoclonal antibody that targets the extracellular domain of EGFR and impedes the interaction of endogenous ligands with it [5]. The sensor cells were individually treated with EGF, TGF-α (100 ng/mL), or Cetuximab (400 nM), or co-treated with EGF and Cetuximab, after which they were investigated by using fluorescence microscopy (Figure 3A). The sensor cells treated with TGF-α showed fluorescence translocation comparable to EGF-treated sensor cells, indicating that TGF-α bound to EGFR and induced dimerization. On the other hand, Cetuximab-treated sensor cells showed negligible fluorescence in the mitochondria (Figure 3A) and Cetuximab also repressed the fluorescence translocation induced by EGF treatment in the co-treated cells (Figure 3A,B). Pearson’s *r* values were 0.1, 0.8, 0.8, 0.1, and 0.1, for the control (untreated), EGF-, TGF-α-, Cetuximab-, and EGF/Cetuximab-treated sensor cells, respectively (Figure 3B). Compared to the control cells, Pearson’s *r* value increased 8-fold when the sensor cells were challenged with EGF or TGF-α but did not show meaningful difference when they were treated with Cetuximab. This result indicates that the developed sensor cells can discriminate between the EGFR agonist and antagonist with excellent specificity.

The formation of CPC reaction products was verified based on Western blot analysis using anti-mCherry antibodies. The sensor cells treated with target ligand EGF or EGFR agonist TGF-α showed the formation of cleavage product whereas the control and Cetuximab-treated sensor cells produced negligible amount of CTC product, as shown in (Figure 3C. When sensor cells were co-treated with EGF and Cetuximab, the amount of CTC product decreased by approximately 80% compared to samples treated with EGF. We observed both the MTS-mCherry (faint) and mCherry bands because MTS was cleaved from the cargo by mitochondrial-processing peptidases after being imported into the mitochondria [53,54]. This result clearly indicates that the specific cleavage of amide bonds and the subsequent activation of MTS were induced by the intein-mediated reaction upon the binding of the target.

### 3.4. Monitoring Ca^2+^ Signaling Induced by EGF Stimuli

Following the detection of EGF binding to EGFR, we further investigated the use of the sensor cells to monitor downstream signaling initiated by ligand binding. The binding of EGF to EGFR activates tyrosine kinase activity that is responsible for a protein phosphorylation cascade associated with a transient increase in cytosolic Ca^2+^ concentration. The nature of the Ca^2+^ response to EGF stimulation is diverse, including the influx of external Ca^2+^ via plasma membrane Ca^2+^ channels and/or depletion of the intracellular Ca^2+^ reservoir involving phosphatidylinositol [55]. The varying nature of the Ca^2+^ response by different cell types is not yet clearly understood. Hence, we investigated Ca^2+^ signaling in EGF-stimulated cells using our previously developed Ca^2+^-responsive sensor cells [39].

**Figure 4:**
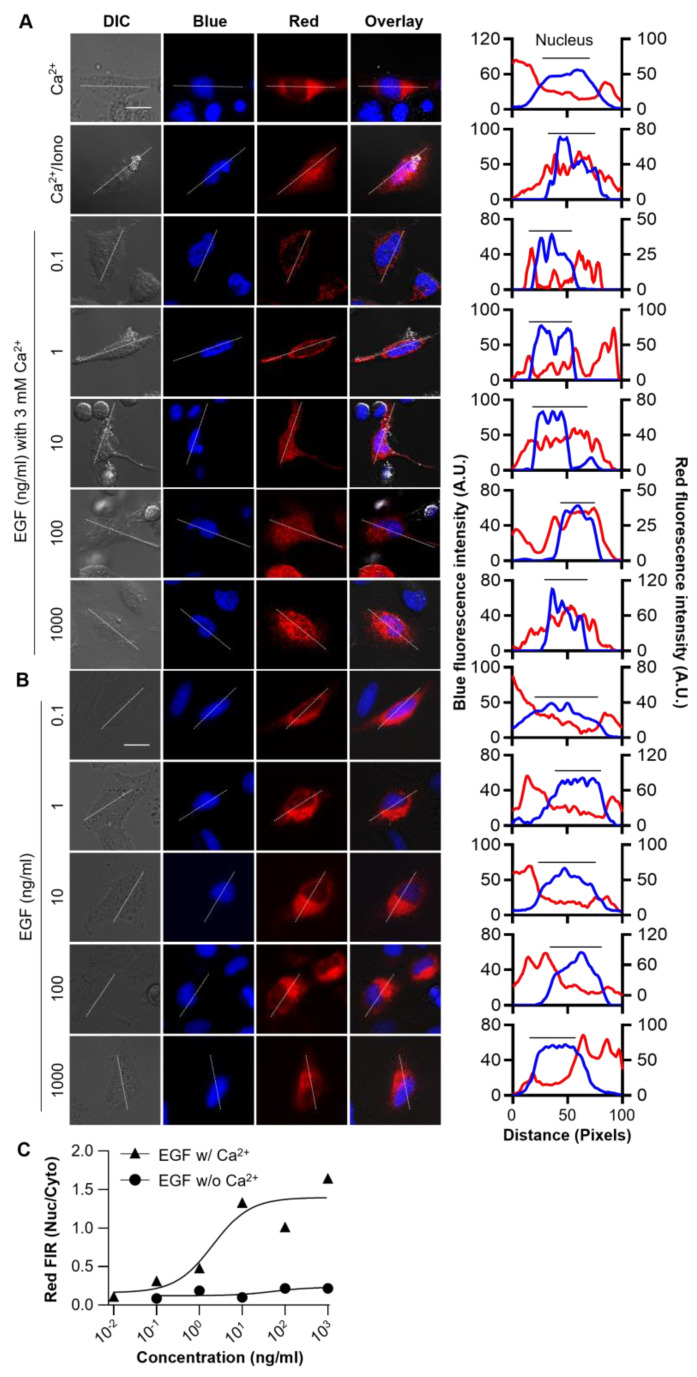
Detection of EGF-mediated Ca^2+^ signaling using the sensor cells. (**A**) Fluorescence images of Ca^2+^-detecting sensor cells treated with 3 mM Ca^2+^ (row 1), 3 mM CaCl_2_ with 20 nM ionomycin (row 2), or varying concentrations of EGF (ranging from 0.1 ng/mL to 1000 ng/mL) in the presence of external Ca^2+^ (rows 3–7). (**B**) Fluorescence images of Ca^2+^-detecting sensor cells treated with varying concentrations of EGF (ranging from 0.1 ng/mL to 1000 ng/mL) in the absence of Ca^2+^ (rows 1–5). The nucleus was stained with Hoechst (blue) (scale bar = 20 µm). EGF-induced Ca^2+^ signaling was observed only in the presence of external Ca^2+^. (**C**) Plot of red fluorescence intensity ratio in nucleus/cytoplasm at different EGF concentration in the presence and absence of Ca^2+^.

Briefly, the Ca^2+^-detecting sensor cells were prepared via genetically encoding two sensor proteins. Each contains a fusion of a calcium-binding domain (CaM or CaMBD), the split-intein of the vacuolar ATPase subunit (VMA^N^ or VMA^C^) of *S. cerevisiae*, and a split nuclear localization signal (NLS^N^ or NLS^C^) peptide with one AFP tag; namely, NLS^N^- VMA^N^-CaM and CaMBD-VMA^C^-NLS^C^-mCherry. The increase in cytosolic Ca^2+^ induces heterodimerization of CaM and CaMBD, which brings two split *VMA* inteins into proximity to trigger the intein-mediated PTS to generate the active NLS peptide. mCherry is used as a translocation reporter. The Ca^2+^-detecting sensor cell was used for the monitoring of EGF activated Ca^2+^ signaling. Ca^2+^-detecting sensor cells were challenged with varying concentration of EGF ranging from 0.1 ng to 1 × 10^3^ ng/mL in the presence or absence of extracellular Ca^2+^. The translocation of red fluorescence signal from cytosol to the nucleus was observed from sensor cells treated with EGF only in the presence of external Ca^2+^ (Figure 4A). The sensor cells treated with EGF in the absence of external Ca^2+^ did not show fluorescence translocation (Figure 4B) suggesting that the EGF-induced Ca^2+^ signaling is originated from the influx of external Ca^2+^ rather than the depletion of internal Ca^2+^ reservoir in this experiment. This result shows that cell-based sensor system can be used to monitor not only the presence of target molecule, EGF, but also to investigate consequent cellular signaling. Dose–response analysis showed that the fluorescence intensity ratio in nucleus to cytosol has increased to over 1 when challenged with above 10 ng/mL concentration of EGF in presence of external Ca^2+^ (Figure 4C).

The cell exposed to high external Ca^2+^ concentration without EGF-stimulation did not show fluorescence translocation. The Ca^2+^-detecting sensor cells were able to report the transient change in Ca^2+^ concentration due to signal accumulation by the irreversible nature of intein-mediated CPS reaction which allows the monitoring of the history of exposure to biologically active molecules. Our result shows that cell-based sensor cells can be used to monitor not only the presence of target molecule, EGF, but also to investigate consequent cellular signaling.

## 4. Conclusions

In this study, we developed a live cell-based biosensor for the monitoring of the EGF-EGFR interaction, which is a crucial biomarker in cancer diagonostics as well as treatments. For the detection of target molecule EGF, we designed a reporter system based on the target-triggered activation of MTS peptides that can transport a fluorescent cargo into the targeted cellular compartment (mitochondria) to convey the presence of the target or the target’s interaction. Co-localization analysis provided a tool for the quantitative assessment of EGF-mediated signaling. The developed sensor cells showed an LOD of 0.1 ng/mL, which is a remarkable sensitivity level for the detection of targets in complex biological samples. Moreover, the developed sensor cells could detect the analytes based on their function rather than their structure, as is often seen in highly sensitive immunosensors. The interaction of agonist TGF-α with EGFR as well as inhibition of the EGF-EGFR interaction by antagonist Cetuximab were monitored using the developed sensor. We then explored the monitoring of EGF-induced Ca^2+^ signaling in sensor cells using a previously reported Ca^2+^-detecting sensor cell to reveal the Ca^2+^ signaling in sensor cells are prompted by the influx of external Ca^2+^. These sensor cells provide the possibility of investigating cell-type-specific EGF Ca^2+^ signaling. With the sensitivity and specificity of the developed sensor cells as well as the capability of investigating the signal cascade following EGF exposure, this cell-based sensor technology provides a crucial tool for drug screening as well as disease diagnosis at an early stage.

## Data Availability

Not applicable.

## Supplementary Materials

The following supporting information can be downloaded at: https://www.mdpi.com/article/10.3390/bios13030383/s1, Figure S1: Tests of designed modified mitochondrial targeting sequence (rMTS) for the sensor cells based on predict programs.
