# Supplementary material for: Cell-Based Sensors for the Detection of EGF and EGF-Stimulated Ca2+ Signaling
Source: Biosensors (Basel). 2023 Mar 14;13(3):383. doi: 10.3390/bios13030383 (PMC10045995; doi:10.3390/bios13030383)
Supplement: Supplementary file 1 [file biosensors-13-00383-s001.zip › biosensors-2211108-supplementary.pdf]

biosensors

Supporting Information

Cell-Based Sensors for the Detection of EGF and EGF-Stimulated Ca^{2+} Signaling

Euiyeon Lee [1,2,†], Keshab Lal Shrestha [1,†], Seonhye Kang [1], Neethu Ramakrishnan [1] and Youngeun Kwon [1,*]

1 Department of Biomedical Engineering, Dongguk University, Seoul 04620, Republic of Korea
2 Department of Chemistry and Chemical Biology, Rutgers University, Piscataway, NJ 08854, USA
* Correspondence: ykwon@dongguk.edu
† These authors contributed equally to this work.

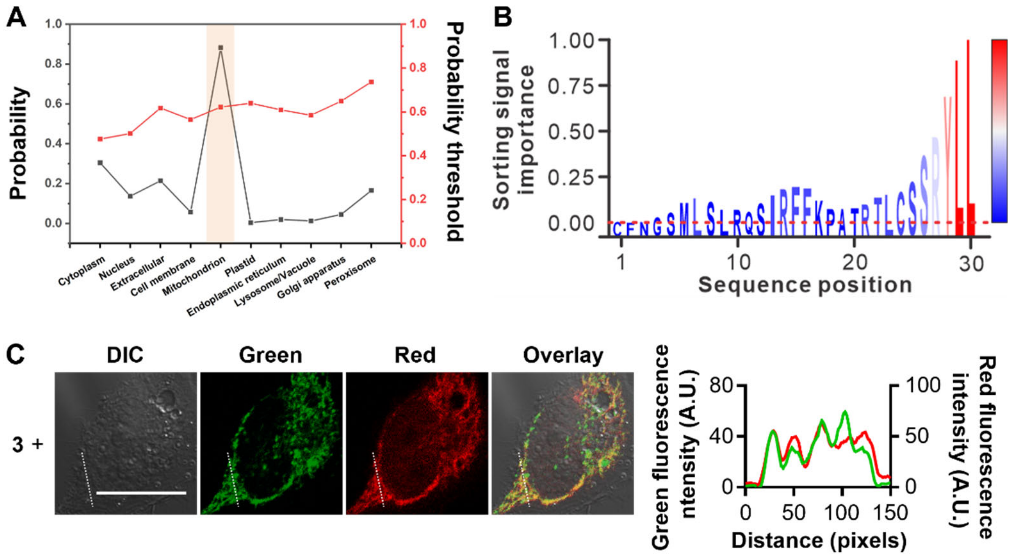

Figure S1. Tests of designed modified mitochondrial targeting sequence (rMTS) for the sensor cells based on predict programs. (**A**) The predicted rMTS location is mitochondria. Probability is compared against probability threshold and only mitochondrial probability is above the probability threshold. (**B**) Sequence LOGOs shows the amino acid sequence of high importance associated with the mitochondrial transit signal among the rMTS sequences. (**C**) The delivery of an AFP into the mitochondria by rMTS was confirmed by monitoring the cellular localization of the fusion protein **3** (scale bar = 20 μm).
